# Coronavirus disease 2019 (COVID-19) in a pregnant women with treatment resistance thrombocytopenic purpura with and suspicion to HELLP syndrome: a case report

**DOI:** 10.1186/s12884-021-04030-x

**Published:** 2021-08-18

**Authors:** Amir Hossein Norooznezhad, Maryam Nurzadeh, Mohammad Hasan Darabi, Mahsa Naemi

**Affiliations:** 1 Medical Biology Research Center, Health Institute Research Center, Kermanshah University of Medical Sciences, Kermanshah, Iran; 2 Maternal, Fetal and Neonatal Research Center, Vali-Asr Hospital, Imam Khomeini Hospital Complexes, Tehran University of Medical Sciences, Tehran, Iran; 3 Department of Obstetrics and Gynecology, Shariati Hospital, Tehran University of Medical Sciences, Tehran, Iran; 4 Fetomaternal Department, Shariati Hospital, Tehran University of Medical Sciences, North-Kargar Avenue, Tehran, Iran

**Keywords:** COVID-19, Pregnancy, Immune thrombocytopenic purpura, Immunosuppression

## Abstract

**Background:**

Coronavirus disease 2019 (COVID-19) still is a global emergency. According to the studies, pregnant women are of the at risk populations and any underlying disease(s) might even worsen their condition. The aim of this study is reporting a complex case of immune thrombocytopenic purpura (ITP) during pregnancy who has been diagnosed with COVID-19 as well as suspicion of HELLP syndrome.

**Case presentation:**

A 24-year-old woman with a platelet count of 6000/mL and resistance to conventional therapies was referred. A day after starting 0.5 g/day of methylprednisolone for her, fever and a decrease in SpO2 presented. According to the paraclinical investigations, COVID-19 was diagnosed and the conventional COVID-19 treatments started for her (the methylprednisolone pulse stopped). Due to the increased liver enzymes and low platelet count, with suspicion of HELLP syndrome, cesarean section surgery was performed which resulted in a healthy neonate. Then, the methylprednisolone pulse was restarted for and she developed an increase in the platelet count.

**Conclusion:**

It is not clear how COVID-19 and pregnancy affected the patient’s condition and the underlying disease; however, it seems the delivery and/or restarting the methylprednisolone pulses caused improvement in her condition.

## Background

Coronavirus disease 2019 (COVID-19) still is a global emergency [1]. So far different systematic reviews and meta-analyses have studied the impact of COVID-19 on pregnant patients [2]. Hematologic consequences of COVID-19 are well documented which some of them such as thrombocytopenia even yielding prognostic values, due to its positive correlation with both disease severity and mortality rate [3, 4]. While the management of immune thrombocytopenic purpura (ITP) in pregnancy is troublesome by itself, thrombocytopenic effects of COVID-19 in a pregnant women with ITP adds more and more to this complexity [5]. Possibly due to lack of evidence, the recommendations derived from the non-pregnant ITP population is applied to pregnant ITP patients with COVID-19, barring some minor precautions. The first-line therapy for these patients is using corticosteroids but it is going to be more complicated in the corticosteroids-resistant treatments [6].

Herein, we would like to explain lessons learned from a pregnant case of ITP with suspicion to HELLP (Haemolysis, Elevated Liver Enzymes, and Low Platelet) syndrome. We believe complicated course of this case can make it a worthwhile addition to literature.

## Case presentation

In April 2020, a 24-year-old primigravida woman at the gestational age of 29 and 1/7 weeks with a history of ITP and splenectomy (11 years ago) was referred to our tertiary level III center. Other than the mentioned condition, she had no underlying disease. The patient was referred from another hospital due to a decreased platelet count with no response to synchronized treatment of prednisolone (5 mg/day) and intravenous immunoglobin (IVIG; 1 g/kg). In ours and the previous center, she revealed no signs or symptoms in favor of SARS-CoV-2 infection and claimed no contact with a confirmed/suspected case of COVID-19. At the time of admission, she had no cough, sore throat, dyspnea, chest pain, rhinorrhea, myalgia, or gastrointestinal symptoms. There was also no sign of elevated body temperature (37.1 °C), tachypnea (respiratory rate: 14 breaths per min), tachycardia (heart rate: 86 per min), or decreased oxygen saturation (SpO_2_: 95%). Laboratory investigations showed a normal leukocyte count accompanied by severe thrombocytopenia (6 × 10^3^/mL). Moreover, hemoglobin, coagulation profile, lactate dehydrogenase level, and kidney function tests were all normal; however, she had slightly elevated liver enzymes (Table 1). Unfortunately, C-reactive protein was not requested for her upon admission. Due to the lack of response to the previous treatments and after consulting with hematology service (considering her clinical and laboratory status), they decided to start methylprednisolone pulse (0.5 g/day). About 24 h later (after the second pulse), the patient presented fever (38 °C) accompanied by SpO_2_ 90% but no other signs or symptoms. Considering this finding and regarding the pandemic, a chest computed tomography (CT) scan (with abdominal shield) was requested immediately which showed bilateral patchy ground-glass opacities and consolidations. Following these findings and after consulting with infectious diseases service, oseltamivir, lopinavir/ritonavir, chloroquine, piperacillin/tazobactam, and azithromycin were started for the patient (methylprednisolone was discontinued after the second pulse) and she was transferred to the COVID-19 ward. Also, the result of reverse transcription polymerase chain reaction (RT-PCR) confirmed SARS-CoV-2 infection on the day after. Due to the increasing pattern of liver enzymes on hospital day (HD) 9 (Table 1) and with the suspicion of HELLP syndrome, the pulse of methylprednisolone and platelet transfusion started for the patient after a consult with hematology service and she underwent cesarean section without any complication. A healthy male neonate with Apgar scores 9 and 10 was born with negative SARS-CoV-2 RT-PCR result. The pulse continued for the next 4 days and discontinued then (HD13). After 4 treatment-free days, the platelet count was 71,000/mL and no pregnancy- nor ITP-related complications were observed. The follow-ups after 6 months revealed no problem at all with both mother and the infant. This case belongs to a research proposal with the ethical approval code of the Medical Ethics Committee of Tehran University of Medical Sciences (IRB: IR.TUMS.VCR.1398.1082).

**Table 1:** Laboratory findings of the patient before and after treatment with methylprednisolone

Variable	Before MP	HD 1 *	HD 9 ⁑	HD 13 ⁑*	HD 17 ⁑⁑
White-cell count (per mL)	9040	10,500	9290	8200	9000
Absolute lymphocyte count (per mL)	361 (4%)	1575 (15%)	650 (7%)	820 (10%)	1440 (16%)
Absolute neutrophil count, (per mL)	8226 (91%)	8505 (81%)	8268 (89%)	7134 (87%)	7470 (83%)
Platelet count× 10^3^ (per mL)	6➔^┼^62	56	6➔^┼┼^105	75	71
Hemoglobin (gr/dL)	11.2	11.6	10.4	9.3	11.5
Hematocrit (%)	34	35	31.3	29.3	36.4
Erythrocyte sedimentation rate (mm/h)	57	N/A	N/A	N/A	N/A
C-reactive protein (mg/L)	N/A	30	N/A	N/A	N/A
Creatinine (mg/dL)	0.72	0.64	0.64	0.64	0.4
Blood urea nitrogen (mg/dL)	9	16	14	26	12
Aspartate aminotransferase (U/L)	53	N/A	346	1173	133
Alanine aminotransferase (U/L)	65	N/A	477	2030	800
Lactate dehydrogenase (U/L)	430	N/A	971	1301	546

*MP* Methylprednisolone pulse, *HD* Hospital day, *N/A* Not applicable

┼: After 1 unit of single donor platelet transfusion. ┼┼: After 10 units of platelet transfusion. *: The patient was transferred to the COVID-19 ward. ⁑: Cesarean section performed and MP started. ⁑*: After the last pulse of MP. ⁑⁑: Four days after last MP pulse. →: following transfusion. During the hospitalization, other laboratory parameters such as levels of total bilirubin, albumin, fibrinogen, prothrombin time (PT), partial thromboplastin time (PTT), Na, and K were in the normal range

## Discussion and conclusion

Herein, we presented a known case of ITP at her 29th week of gestational who was referred to our center with a platelet count of 6 × 10^3^/mL during the COVID-19 pandemic. The patient received methylprednisolone pulses and not long after, she developed fever, seemingly the first sign of COVID-19 infection. The uncertainty of immunosuppressive therapy at that time led us to stop the corticosteroid (according to national April 2020 guideline) and start the conventional COVID-19 treatment. Abnormal patterns of liver function tests in accompanied with low platelet count made us to consider HELLP syndrome as a probable diagnosis. Therefore, patient underwent an emergency caesarian section on the HD 9 and received her next five pulses of methylprednisolone. After four treatment-free days, liver enzymes decreased and platelet count increased.

Gestational thrombocytopenia (mostly with a platelet count between 130 and 150 × 10^3^/mL) might be seen during pregnancy [7] as the second frequent hematologic-related change of gestation, after anemia [8]. Very low platelet count and response to corticosteroids make this diagnosis very unlikely in our patient [7]. Thrombocytopenia itself is a complication of COVID-19 and is a heralding sign of poor prognosis [3]. Papageorghiou et al., concluded that there is a strong association between COVID-19 and preeclampsia [9]. Thus, COVID-19 might have played a significant role in reducing our patient’s already low platelet counts either via COVID-19 induced thrombocytopenia or by causing preeclampsia at its most severe form (i.e., HELLP syndrome).

Differentiation between these two etiologies would be extremely difficult as both of them can cause elevated liver enzymes, elevated LDH, elevated BUN/Cr ratio and of course thrombocytopenia [10]. Hypertension and proteinuria will turn the tide in favor of a HELLP syndrome diagnosis but atypical cases of HELLP syndrome who are normotensive and without proteinuria should not be overlooked [11]. We believe a dramatic response to delivery makes HELLP syndrome more likely in our patient, but as mentioned before it is highly speculative.

Nesr et al. [5], have reported a similar case which was a 34-year-old woman previously diagnosed with ITP who presented with dry cough, fever, petechial and gingival bleeding at her 24th week of gestation. Considering the platelet count of 13 × 10^3^/mL, she received intravenous immunoglobulin (1 g/kg) and oral prednisolone (1 mg/ kg) with favorable results. Also, just like our case, they reported lymphopenia in their patient, too [5].

A dark spot of this report is the synchronism of methylprednisolone pulse treatment and COVID-19 manifestation (with a short delay). Considering the emerged median duration of the COVID-19 incubation period (5.1 days) [12] this question might rise that if the high-dose Immunosuppressives could decrease this period or not. We wish that the patient was evaluated for COVID-19 infection before the methylprednisolone pulse. Also, early request of erythrocyte sedimentation rate (ESR), C-reactive protein (CRP), and absolute lymphocyte count could have provided us with some early indicators of COVID-19 infection. Although as mentioned in the case presentation, there were no apparent clinical indications for requesting such tests.

This report presented a pregnant woman with ITP who developed some features of HELLP syndrome during hospitalization which led us to consider emergency delivery. This report can provide us with invaluable information on corticosteroid therapy in the treatment of pregnant women with thrombocytopenia and COVID-19 infection. The physicians should be aware of COVID-19 induced thrombocytopenia and its differential diagnoses, especially in pregnant patients where finding out the exact diagnosis will be much more difficult than usual and if they could not distinguish between etiologies of thrombocytopenia, it is better to consider the worst-case scenario in order to prevent any unwanted outcome, just like our case.

## Data Availability

Data would be available at online requests from Dr. Mahsa Naemi, the corresponding author.
